# HOXA3 Knockdown Impairs hESC-Derived Thymic Epithelial-like Cell Differentiation and CXCL12-Associated Early T-Lineage Support

**DOI:** 10.3390/cells15191764

**Published:** 2026-09-27

**Authors:** Huanhuan Shan, Yingjie Fu, Xinyi Shi, Sen Chen, Yuyou Duan

**Affiliations:** 1 Laboratory of Stem Cells and Translational Medicine, Center for Medical Research on Innovation and Translation, Institute of Clinical Medicine, The Second Affiliated Hospital, School of Medicine, South China University of Technology, Guangzhou 510006, China; 2 Laboratory of Stem Cells and Translational Medicine, Institute for Life Science, School of Medicine, South China University of Technology, Guangzhou 510006, China; 3 National Engineering Research Center for Tissue Restoration and Reconstruction, South China University of Technology, Guangzhou 510006, China; 4 The Innovation Centre of Ministry of Education for Development and Diseases, The Second Affiliated Hospital, School of Medicine, South China University of Technology, Guangzhou 510006, China

**Keywords:** HOXA3, thymic epithelial-like cell, human embryonic stem cells, CXCL12, early T-cell differentiation, thymic epithelial differentiation

## Abstract

**Highlights:**

**What are the main findings?**

- HOXA3 knockdown during the third pharyngeal pouch endoderm-to-thymic epithelial-like cell transition reduced thymic epithelial-associated features and was accompanied by broad transcriptomic remodeling in hESC-derived cultures.
- HOXA3 knockdown reduced CXCL12 expression and was accompanied by HOXA3 ChIP enrichment at a predicted CXCL12 regulatory region; CXCL12 partially restored CD45^+^CD3^+^ early T-lineage output in knockdown co-cultures.

**What are the implications of the main findings?**

- These findings support involvement of HOXA3 in the third pharyngeal pouch endoderm-to-thymic epithelial-like transition in this hESC model.
- The study links HOXA3 knockdown with reduced CXCL12 expression and diminished early T-lineage support, with CXCL12 supplementation indicating a contributory role.

**Abstract:**

Thymic epithelial cells (TECs) provide essential signals for T-cell development, yet the mechanisms governing human TEC differentiation and the acquisition of T-cell-supporting function remain incompletely understood. Using a three-stage system that directs human embryonic stem cells (hESCs) through definitive endoderm and third pharyngeal pouch endoderm (3PPE) toward a thymic epithelial-like lineage, we investigated the role of HOXA3 during thymic epithelial-like cell (TEC-like cell) differentiation. HOXA3 knockdown during 3PPE-to-TEC-like cell transition impaired thymic epithelial differentiation, as indicated by reduced K8, AIRE, and CD205 expression and decreased FOXN1 protein. HOXA3-knockdown TEC-like cells also showed a markedly reduced capacity to support the generation of CD45^+^CD3^+^ cells from cord blood CD34^+^ progenitors. Bulk RNA sequencing identified 1040 upregulated and 774 downregulated genes, revealing broad remodeling of cell-cycle, motility, chemotaxis, angiogenesis, and tissue-remodeling programs. CXCL12 was among the downregulated genes, and its reduction was confirmed at both mRNA and protein levels. Motif analysis identified a putative HOXA3-binding site, and conventional ChIP-PCR showed enrichment of the predicted CXCL12 regulatory region in HOXA3 immunoprecipitates. Recombinant CXCL12 significantly increased CD45^+^CD3^+^ cell generation in HOXA3-knockdown co-cultures. Together, these data support an association between HOXA3 knockdown, reduced acquisition of a thymic epithelial-like phenotype, and diminished early T-lineage support, with CXCL12 partially accounting for the functional phenotype. This study provides a tractable hESC-based model for studying human thymic epithelial development and TEC-mediated early T-lineage differentiation.

## 1. Introduction

The thymus provides a specialized microenvironment for the generation of a diverse and self-tolerant T-cell repertoire, a process that depends on coordinated interactions between developing thymocytes and thymic epithelial cells (TECs) [1,2]. TECs comprise functionally distinct cortical (cTEC) and medullary (mTEC) compartments that support successive stages of thymocyte development and collectively mediate positive and negative selection [3,4]. Disruption of thymic epithelial integrity, including that associated with age-related thymic involution, compromises thymopoiesis and immune competence [5,6]. Despite their central role, the molecular mechanisms governing human TEC differentiation and linking thymic epithelial lineage acquisition to T-cell-supporting function remain incompletely defined [7].

Human embryonic stem cell (hESC)-based differentiation systems provide experimentally tractable models for recapitulating key stages of human thymic epithelial development [8,9,10]. During embryogenesis, thymic epithelial progenitors arise from the endodermal epithelium of the third pharyngeal pouch (3PPE) and subsequently differentiate toward the thymic epithelial lineage [11]. By sequentially recapitulating definitive endoderm induction, 3PPE specification, and thymic epithelial differentiation, these systems enable stage-specific interrogation of the molecular programs that govern human TEC development [12]. However, the transcriptional regulators controlling the 3PPE-to-TEC transition, and how this developmental program is coupled to the acquisition of T-cell-supporting function, remain incompletely defined.

HOXA3 is a developmental homeobox transcription factor with established roles in embryonic patterning and pharyngeal organogenesis [13,14]. Genetic studies in mice have shown that HOXA3 is required for pharyngeal apparatus and thymus development, including the differentiation and morphogenesis of thymic epithelial progenitors [14,15]. In our previous study, we further demonstrated that HOXA3 regulates the generation of hESC-derived 3PPE through EPHB2-mediated Wnt signaling [16]. However, the transcriptional mechanisms governing the subsequent 3PPE-to-TEC transition remain incompletely defined [17], and whether HOXA3 continues to function after 3PPE specification is unknown. Beyond acquiring thymic epithelial identity, developing TECs must establish stromal signals that support hematopoietic progenitor migration and T-cell development [18]. Among these signals, CXCL12-CXCR4 signaling regulates progenitor localization and intrathymic T-cell development [19,20]. Whether CXCL12 is a direct transcriptional target of HOXA3 and contributes to the acquisition of T-cell-supporting capacity by developing human TECs has not been determined.

In this study, we used a three-stage hESC differentiation system to examine the consequences of HOXA3 knockdown during the 3PPE-to-thymic epithelial-like transition and for the early T-lineage-associated output of derived stromal cultures. HOXA3 knockdown reduced thymic epithelial-associated markers and FOXN1 protein and was associated with fewer CD45^+^CD3^+^ cells in co-culture with human cord blood CD34^+^ hematopoietic progenitors. Bulk transcriptomic analysis revealed broad molecular remodeling following HOXA3 knockdown and identified CXCL12 among the downregulated genes. Motif analysis identified a putative HOXA3-binding site, while conventional ChIP-PCR showed enrichment of the predicted CXCL12 regulatory region in HOXA3 immunoprecipitates. Recombinant CXCL12 partially increased CD45^+^CD3^+^ cell generation in HOXA3-knockdown co-cultures. Accordingly, the present data support involvement of HOXA3 in this hESC-derived thymic epithelial-like differentiation model and a contributory role for CXCL12 in the associated early T-lineage phenotype.

## 2. Materials and Methods

### 2.1. Culture and Maintenance of hESCs

hESCs (H9 line, WA09) were obtained from the WiCell Research Institute (Madison, WI, USA) under Material Transfer Agreements (Nos. 19-W0512, 24-W0162, and 24-W0163) and were cultured on mitotically inactivated mouse embryonic fibroblast (MEF) feeder layers. The culture medium consisted of DMEM-F12 (Gibco, Grand Island, NY, USA) supplemented with 20% KnockOut™ Serum Replacement (Thermo Fisher Scientific), 4 ng/mL basic fibroblast growth factor (bFGF, Peprotech, Rocky Hill, NJ, USA), 100 mM L-glutamine (Gibco), 1% MEM non-essential amino acids (Thermo Fisher Scientific, Waltham, MA, USA), and 0.1 mM β-mercaptoethanol (Sigma-Aldrich Co. LLC, Saint Louis, MO, USA). The medium was replaced daily.

### 2.2. Human Umbilical Cord Blood

Human umbilical cord blood samples were collected from donors with normal full-term deliveries after obtaining written informed consent. The collection and use of cord blood samples were approved by the Ethics Committee of Guangzhou First People’s Hospital (Approval No. K-2021-008-02) and were performed in accordance with the Declaration of Helsinki.

### 2.3. In Vitro Differentiation of hESCs into TEC-like Cells

For directed differentiation of H9 hESCs into TEC-like cells, we adopted a previously established stepwise protocol [16]. Briefly, H9 cells were first cultured on gelatin-coated plates with a feeder layer of mitotically inactivated MEFs for 5 days. To induce DE, the medium was switched to X-VIVO 20 (Lonza, Basel, Switzerland) containing 100 ng/mL Activin A (Peprotech), 50 ng/mL WNT3A (Peprotech) or 3 µM CHIR99021 (Selleck Chemicals, Houston, TX, USA), and 10 µM Y-27632 (Selleck) for the first day, followed by X-VIVO 20 supplemented with 100 ng/mL Activin A and 0.5% B27 (Gibco) from day 2 onward. At day 4, the resulting endodermal cells were dissociated with TrypLE (Gibco) and replated on Matrigel (Corning, Corning, NY, USA)-coated plates in DMEM-F12 or EGM2 (Lonza) medium supplemented with 0.5% B27, 1 µM retinoic acid (Sigma), 10 µM SB431542 (MedChemExpress, Monmouth Junction, NJ, USA), 20 ng/mL BMP4 (Peprotech), and 2.5 µM IWP2 or IWR1 (Selleck) to promote 3PPE specification. This medium was replaced daily until day 9. For terminal differentiation of 3PPE into TECs, cells were cultured from day 9 onward in DMEM-F12 containing 50 ng/mL WNT3A, 10 ng/mL BMP4, 50 ng/mL FGF8, 5 ng/mL FGF10 (all from Peprotech), 10 ng/mL heparin sodium (GlpBio, Montclair, CA, USA), 10 µM SB431542, 2.5 µM SANT-1 (Selleck), 50 µg/mL 2-phosphate-L-ascorbic acid trisodium salt (Sigma), 0.5% B27, and 1 µM RA for 4 days, with daily medium changes.

### 2.4. Lentiviral shRNA-Mediated HOXA3 Knockdown

Lentiviral vectors encoding short hairpin RNA (shRNA) targeting human HOXA3 (shHOXA3, target sequence: 5′-CGGTGGCTATCTGAACTCTAT-3′) and a non-targeting control shRNA (shControl, target sequence: 5′-TTCTCCGAACGTGTCACGT-3′) were obtained from Guangzhou IGE Biotechnology Co., Ltd. (Guangzhou, China). Lentiviral particles were produced by co-transfecting HEK293T cells with the shRNA plasmid and packaging plasmids (pMD2.G and psPAX2) using PEI (Polysciences, Warrington, PA, USA) according to the manufacturer’s instructions. Viral supernatants were collected at 48 h post-transfection, filtered through 0.45-μm pore-size filters, and concentrated by ultracentrifugation at 25,000× *g* for 2 h at 4 °C. The viral pellets were resuspended in sterile PBS and stored at −80 °C until use.

For lentiviral transduction, 3PPE-stage cells were seeded on day 9 and transduced with lentivirus after attachment (approximately 24 h after seeding) in the presence of 4 μg/mL polybrene (Sigma-Aldrich). At 24 h post-transduction, the medium was replaced with fresh differentiation medium. Cells were harvested 72 h after transduction (day 13) for downstream analyses, including RT-qPCR, Western blotting, immunofluorescence, and co-culture experiments. The same post-transduction endpoint (72 h, day 13) was used for the HOXA3-knockdown analyses described below.

### 2.5. RNA Extraction and Quantitative Real-Time PCR (RT-qPCR)

Total RNA was extracted from cells at the indicated stages using TRIzol reagent (Takara Bio, Kusatsu, Shiga, Japan) according to the manufacturer’s instructions. RNA concentration and purity were assessed using a NanoDrop 2000 spectrophotometer (Thermo Fisher Scientific). For reverse transcription, 1 μg of total RNA was converted to cDNA using the HiScript III RT SuperMix for qPCR (+gDNA wiper) (Vazyme Biotech, Nanjing, China) following the manufacturer’s protocol. Quantitative real-time PCR was performed using ChamQ Universal SYBR qPCR Master Mix (Vazyme) on a QuantStudio system (Applied Biosystems, Foster City, CA, USA). GAPDH was used as an internal control, and relative gene expression was calculated using the 2^−ΔΔCt^ method. All primer sequences are listed in Supplementary Table S1.

### 2.6. Immunofluorescence Staining

For immunofluorescence staining, cells were seeded onto coverslips in 12-well plates one day prior to fixation. After washing with PBS, cells were fixed with 4% paraformaldehyde (PFA, Biosharp, Hefei, Anhui, China) for 15 min at room temperature, permeabilized with 0.3% Triton X-100 (Solarbio, Beijing, China) for 20 min, and blocked with 5% normal goat serum (Boster, Wuhan, Hubei, China) for 1 h at room temperature. Cells were then incubated with primary antibodies overnight at 4 °C. The following day, after rewarming at 37 °C for 1 h and washing three times with PBS, cells were incubated with appropriate fluorescently labeled secondary antibodies for 2 h at room temperature in the dark. Nuclei were counterstained with DAPI (Beyotime, Biotechnology, Shanghai, China) for 10 min at room temperature in the dark, and coverslips were mounted onto glass slides using an anti-fade mounting medium (Beyotime, China). Images were acquired using a Zeiss 880 multiphoton laser scanning microscope (Zeiss, Oberkochen, Germany) or a Nikon single-photon confocal microscope (Nikon, Tokyo, Japan). All antibodies used in this study are listed in Supplementary Table S2.

### 2.7. Flow Cytometry

Cells were collected and washed twice with cold PBS. For cell surface marker staining, cells were stained with fluorochrome-conjugated antibodies against CD45 and CD3 and incubated at 4 °C in the dark for 30 min. DAPI was added immediately before acquisition to eliminate dead cells. Flow cytometry was performed on a BD Fortessa (BD Biosciences, Franklin Lakes, NJ, USA). FlowJo (V10) software was used for data analysis. For gating, debris was first excluded by FSC-A/SSC-A, doublets were excluded by FSC-A/FSC-H and SSC-A/SSC-H, and dead cells were excluded using DAPI. The CD45^+^CD3^+^ population was defined as viable single cells that were CD45^+^ and CD3^+^.

### 2.8. Western Blotting

Cells were lysed by sonication in RIPA lysis buffer (Beyotime) supplemented with protease inhibitor cocktail (Beyotime) and PMSF (Beyotime). Lysates were centrifuged at 14,000× *g* for 15 min at 4 °C, and supernatants were collected as total protein extracts. Protein concentrations were determined using a BCA protein assay kit (Boster) according to the manufacturer’s instructions. Equal amounts of protein (20 μg per lane) were separated by 10% SDS-PAGE and transferred onto PVDF membranes (Merck Millipore, Burlington, MA, USA). Membranes were blocked with 5% non-fat milk (Solarbio) in TBST (Tris-buffered saline containing 0.1% Tween-20) for 1 h at RT, followed by overnight incubation at 4 °C with primary antibodies. After washing three times with TBST, membranes were incubated with HRP-conjugated secondary antibodies for 1 h at RT. Protein bands were visualized using an enhanced chemiluminescence (ECL) kit (Affinity Biosciences, Cincinnati, OH, USA) and imaged using a chemiluminescence imaging system. All antibodies used in this study are listed in Supplementary Table S2.

### 2.9. Chromatin Immunoprecipitation Assay

ChIP assays were performed using the ChIP Assay Kit (Beyotime). Briefly, TECs were cross-linked with 1% formaldehyde for 10 min at 37 °C, quenched with glycine, and lysed in SDS lysis buffer containing PMSF and protease inhibitors. Chromatin was sheared by sonication to 200–500 bp fragments, verified by agarose gel electrophoresis. After centrifugation, the supernatant was diluted and 5% was saved as input. Chromatin was pre-cleared with protein A/G magnetic beads, then incubated overnight at 4 °C with 4 μg of anti-HOXA3 antibody (Santa Cruz, Biotechnology, Dallas, TX, USA) or normal mouse IgG (Beyotime) as a negative control. Immune complexes were captured with beads, washed, eluted, and reverse-crosslinked overnight at 65 °C with proteinase K. Purified DNA was PCR-amplified with primers specific for the CXCL12 promoter region (Supplementary Table S3), resolved on 2% agarose gels, and visualized under UV.

### 2.10. Co-Culture of TEC-like Cells with CD34^+^ Hematopoietic Progenitor Cells

Human CD34^+^ hematopoietic progenitor cells were isolated from umbilical cord blood using the Dynabeads™ CD34 Positive Isolation Kit (Thermo Fisher Scientific, Cat. No. 11301D) according to the manufacturer’s instructions. Briefly, mononuclear cells were first isolated by density gradient centrifugation using Ficoll-Paque PLUS (Thermo Fisher Scientific), followed by positive selection of CD34^+^ cells with the Dynabeads™ system. After isolation, CD34^+^ cells were released from the magnetic beads using DETACHaBEAD™ reagent (Thermo Fisher Scientific) and washed prior to use. The purity of isolated CD34^+^ cells, routinely >95%, was verified by flow cytometry. Baseline CD3 staining of the starting CD34^+^ fraction was not included in the current dataset; accordingly, the co-culture assay is interpreted as an early T-lineage-associated readout rather than definitive proof of de novo mature T-cell generation.

For functional assessment, shControl or shHOXA3 iTECs were seeded in 12-well plates and cultured until differentiation was complete. The medium was then replaced with T-cell differentiation medium consisting of RPMI 1640 supplemented with 1× B-27™ Supplement (Thermo Fisher Scientific), 10 ng/mL recombinant human stem cell factor (SCF; Peprotech, Cat. No. 300-07), 5 ng/mL recombinant human interleukin-7 (IL-7; Peprotech, Cat. No. 200-07), 5 ng/mL recombinant human Fms-related tyrosine kinase 3 ligand (FLT3L; Peprotech, Cat. No. 300-19), 10 ng/mL recombinant human Delta-like protein 4 (DLL4; Peprotech, Cat. No. 140-07), and 30 µM L-ascorbic acid-2-phosphate trisodium salt (vitamin C; Sigma-Aldrich, Cat. No. 49752). Purified CD34^+^ cells (1 × 10^5^ cells/well) were then directly added to the iTEC-containing wells. Co-cultures were maintained for one week, with half-medium changes performed every three days.

For rescue experiments, recombinant human CXCL12 (rhCXCL12; MedChemExpress, Cat. No. HY-P70469) was added to the co-culture system at a concentration of 100 ng/mL at the start of co-culture and replenished at each medium change, along with fresh cytokines.

### 2.11. Bulk RNA Sequencing (RNA-Seq)

Total RNA was extracted from Day 13 TEC-like cells derived from shControl and shHOXA3 cultures, with three independent biological replicates per group. For each sample, 100 ng of total RNA was used for library preparation following the ligation-mediated sequencing (LM-Seq) protocol as previously described. Libraries were quantified using a Qubit 2.0 fluorometer (Thermo Fisher Scientific), and their quality and fragment-size distribution were assessed using an Agilent 2100 Bioanalyzer (Agilent Technologies, Santa Clara, CA, USA). The libraries were then multi-plexed, diluted to a final concentration of 2.5 nM, and subjected to single-end sequencing on an Illumina NovaSeq 6000 platform (Illumina, San Diego, CA, USA).

Raw sequencing reads were aligned to the human reference genome (GRCh38) using STAR. Across the six RNA-seq libraries, 37.4–48.7 million clean reads were obtained per sample, with Q30 values ranging from 96.72% to 97.19%. The overall mapping rates ranged from 95.48% to 97.39%, with uniquely mapped reads accounting for 90.68–94.34% of total reads. Gene-level count data were analyzed using DESeq2. Genes with counts ≥ 10 in at least three samples were retained for differential expression analysis. Principal component analysis (PCA) was performed on variance-stabilized expression data generated using the variance-stabilizing transformation (VST) implemented in DESeq2. Differentially expressed genes (DEGs) between shHOXA3 and shControl cultures were identified using an adjusted *p* value < 0.05 and |log_2_(fold change)| > 1.

Functional enrichment analyses were performed using the clusterProfiler R package. Ensemble gene identifiers were converted to Entrez Gene identifiers using org.Hs.eg.db and AnnotationDbi. Gene Ontology (GO) Biological Process enrichment analysis was performed using enrichGO, and Kyoto Encyclopedia of Genes and Genomes (KEGG) pathway enrichment analysis was performed using enrichKEGG with the human organism setting (“hsa”). Upregulated and downregulated DEGs were analyzed separately, using all genes included in the differential expression analysis as the background gene set. *p* values were adjusted for multiple comparisons using the Benjamini–Hochberg method. Enrichment analyses used a *p*-value cutoff of 0.05 and a q-value cutoff of 0.20, with gene-set sizes restricted to 10–500 genes. Enrichment results were ranked and visualized according to gene ratio or enrichment score and adjusted *p* value, as appropriate.

The RNA-seq data have been deposited in the Gene Expression Omnibus under accession number GSE344232.

### 2.12. Statistical Analysis

Each biological replicate represents an independent differentiation experiment starting from H9/WA09 hESC cultures, performed at a different time and from a different passage of the same cell line, rather than repeated measurements from the same differentiation. All quantitative experiments were analyzed using the biological replicate as the statistical unit (*n* = 3 per group unless otherwise indicated), and results are presented as mean ± standard error of the mean (SEM), with individual data points overlaid where applicable. Statistical analyses were conducted using GraphPad Prism 9.5 (Dotmatics, Boston, MA, USA). For RNA-seq data, differential expression analysis was performed using DESeq2 (v1.38.3), and *p* values were adjusted for multiple comparisons using the Benjamini–Hochberg method. Genes with an adjusted *p* value < 0.05 and |log_2_(fold change)| > 1 were considered differentially expressed. For all other quantitative assays—including qRT-PCR, Western blotting, immunofluorescence, and flow cytometry-based T-cell differentiation assays—comparisons between shControl and shHOXA3 groups were analyzed using unpaired, two-tailed Student’s *t*-test. Formal normality testing was not performed because *n* = 3 per group is too small to provide a reliable assessment of the underlying data distribution. The unpaired, two-tailed Student’s *t*-test was used for these pairwise comparisons because the statistical units were independent biological replicates and the readouts were continuous quantitative measurements. Given the small sample size and the inability to robustly verify the normality assumption, the resulting *p* values should be interpreted cautiously together with the individual data points and the magnitude and direction of the observed differences. A *p*-value < 0.05 was considered statistically significant. Significance levels are denoted as follows: * *p* < 0.05, ** *p* < 0.01, *** *p* < 0.001, **** *p* < 0.0001; n.s., not significant.

## 3. Results

### 3.1. Stepwise Differentiation of hESCs Toward TEC-like Cells Through Definitive Endoderm and 3PPE Intermediates

**Figure 1:**
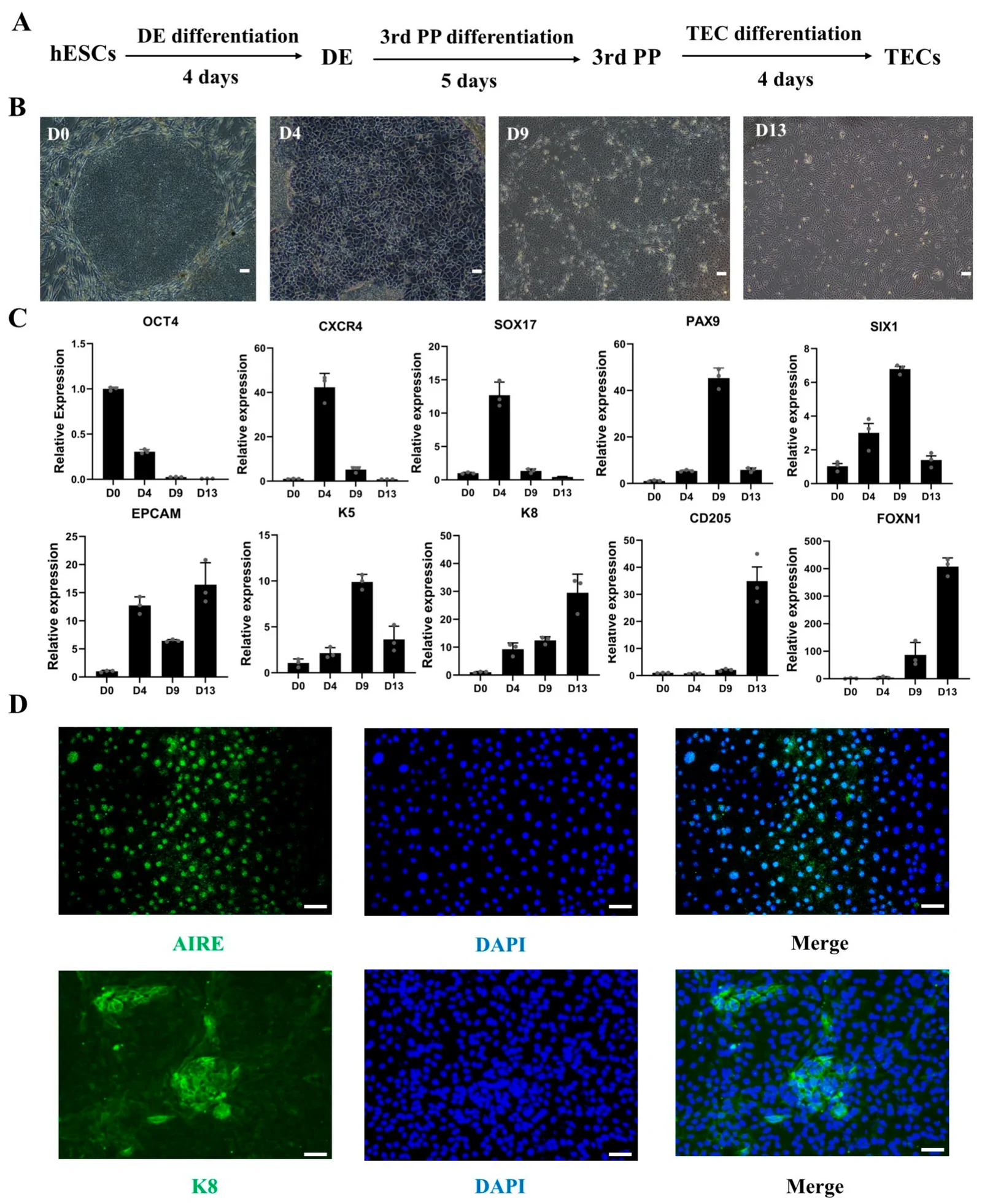
Stepwise differentiation of hESCs toward TEC-like cells through definitive endoderm and 3PPE intermediates. (**A**) Schematic illustration of the three-stage differentiation protocol. Definitive endoderm (DE) was induced during days 1–4, followed by third pharyngeal pouch endoderm (3PPE) specification during days 4–9 and differentiation toward the thymic epithelial-like lineage during days 9–13. (**B**) Representative phase-contrast images showing morphological changes during differentiation at days 0, 4, 9, and 13. Scale bars, 100 μm. (**C**) RT-qPCR analysis of stage-associated genes at days 0, 4, 9, and 13. Expression of the pluripotency-associated gene OCT4; definitive endoderm-associated genes SOX17 and CXCR4; pharyngeal endoderm/3PPE-associated genes PAX9 and SIX1; and epithelial or thymic epithelial-associated genes EPCAM, K5, K8, CD205, and FOXN1 was assessed. Data are presented as mean ± SEM from three independent experiments. (**D**) Representative immunofluorescence images showing AIRE or K8 expression in separate day 13 cultures. AIRE and K8 are shown in green, and nuclei were counterstained with DAPI in blue. Scale bars, 50 μm.

To model human thymic epithelial differentiation in vitro, we used our previously established three-stage protocol [16] to direct hESCs sequentially through definitive endoderm (DE), third pharyngeal pouch endoderm (3PPE), and thymic epithel-like cell differentiation (Figure 1A). DE was induced during days 1–4, followed by 3PPE specification during days 4–9 and differentiation toward the thymic epithelial-like lineage during days 9–13. Cells underwent progressive morphological changes over the 13-day differentiation course (Figure 1B). Undifferentiated hESC colonies gradually acquired an epithelial appearance during DE induction. By day 9, the cultures formed densely organized epithelial colonies, whereas day 13 cultures displayed a predominantly adherent, cobblestone-like morphology consistent with further epithelial differentiation.

To characterize developmental progression, we examined stage-associated gene expression at days 0, 4, 9, and 13 by RT-qPCR (Figure 1C). Pluripotent gene OCT4 expression was highest in undifferentiated hESCs and declined upon differentiation. SOX17 and CXCR4 peaked at day 4, consistent with DE induction, whereas PAX9 and SIX1 were enriched at day 9, supporting the acquisition of a pharyngeal endoderm/3PPE-associated program. During the subsequent transition toward the thymic epithelial lineage, EPCAM was induced and remained elevated at day 13, while K8 and FOXN1 progressively increased and CD205 was strongly induced by day 13. K5 showed a transient increase at day 9 and remained detectable at day 13. The marked induction of FOXN1, a central regulator of thymic epithelial development [21], together with these stage-associated changes, supported acquisition of a thymic epithelial-like molecular program.

Consistent with the transcriptional profile, immunofluorescence staining independently detected AIRE- and K8-expressing cells in day 13 cultures (Figure 1D). Therefore, these findings show that the three-stage protocol recapitulates key features of sequential DE and 3PPE intermediates followed by the acquisition of thymic epithelial-associated characteristics, thereby providing a stage-resolved platform for investigating molecular regulation of the 3PPE-to-TEC-like cell transition.

### 3.2. HOXA3 Knockdown Impairs TEC-like Cell Differentiation from hESCs

**Figure 2:**
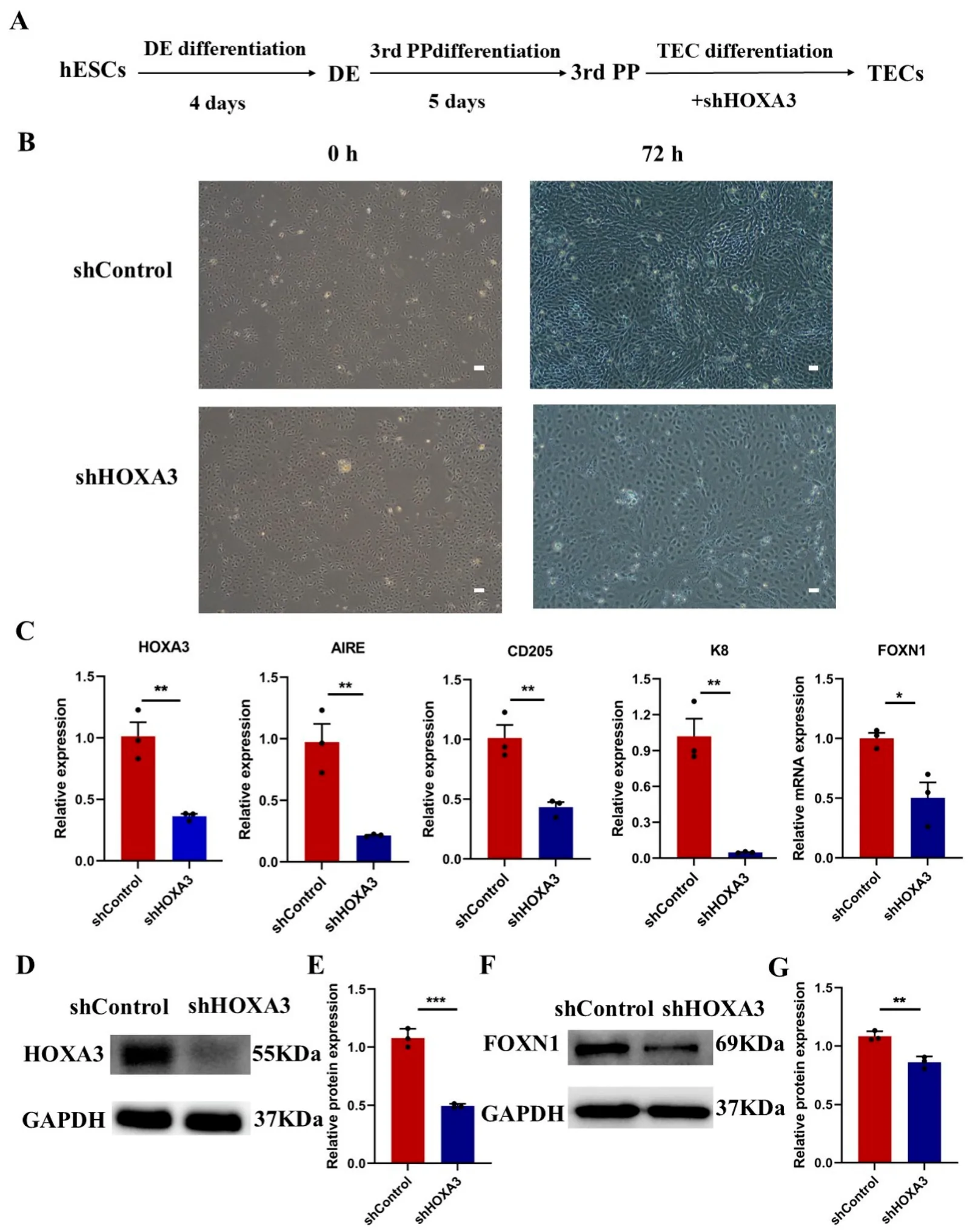
HOXA3 knockdown impairs TEC-like cell differentiation from hESCs. (**A**) Schematic illustration of the shRNA-mediated HOXA3 knockdown strategy. Day 9 3PPE-stage cultures were transduced with a lentiviral shRNA targeting HOXA3 (shHOXA3) or a non-targeting control shRNA (shControl) and analyzed 72 h after transduction. (**B**) Representative phase-contrast images of shControl and shHOXA3 cultures at 0 and 72 h after the transduction. Scale bars, 100 μm. (**C**) RT-qPCR analysis of *HOXA3*, *AIRE*, *CD205*, *K8* and *FOXN1* transcript levels in shControl and shHOXA3 cultures at the experimental endpoint. Data are presented as mean ± SEM from three independent experiments. * *p* < 0.05 and ** *p* < 0.01 versus shControl. (**D**–**G**) Representative Western blots showing HOXA3 (**D**) and FOXN1 (**F**) protein expression in shControl and shHOXA3 cultures. GAPDH was used as a loading control. Densitometric quantification of HOXA3 (**E**) and FOXN1 (**G**) protein levels normalized to GAPDH. Data are presented as mean ± SEM from three independent experiments. ** *p* < 0.01 and *** *p* < 0.001 versus shControl.

To examine the consequences of HOXA3 depletion during the 3PPE-to-thymic epithe-lial-like transition, 3PPE-stage cultures were transduced with a lentiviral shRNA targeting HOXA3 (shHOXA3) or a non-targeting control shRNA (shControl) and analyzed 72 h after transduction (Figure 2A). At the endpoint, shControl cultures displayed an organized cobblestone-like epithelial morphology during TEC-like cell differentiation, whereas shHOXA3 cultures appeared flatter and more irregular and showed reduced cell density (Figure 2B). RT-qPCR efficiently confirmed HOXA3 knockdown and demonstrated significantly reduced expression of the TEC-associated genes K8, AIRE, CD205 and FOXN1 in shHOXA3 cultures compared with shControl cultures (Figure 2C). Representative Western blots showed lower protein signals of HOXA3 and FOXN1, a central regulator of thymic epithelial development [21], in shHOXA3 cultures (Figure 2D–G). These findings indicate impaired acquisition of thymic epithelial-associated features in the HOXA3-knockdown condition.

### 3.3. HOXA3 Knockdown Reduces the Early T-Cell-Supporting Capacity of hESC-Derived TEC-like Cells

**Figure 3:**
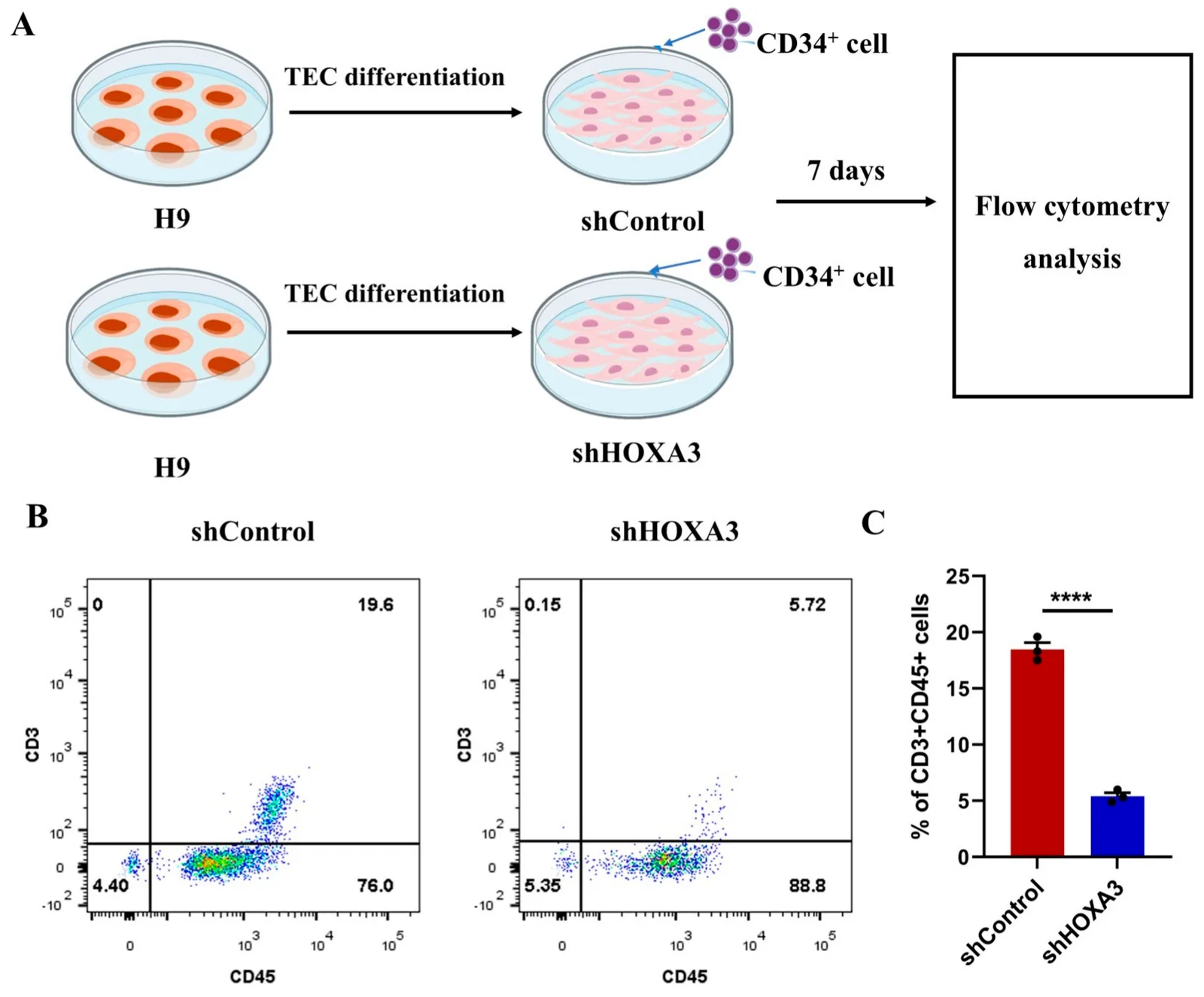
HOXA3 knockdown is associated with reduced CD45^+^CD3^+^ early T-lineage output in co-culture. (**A**) Schematic illustration of the co-culture experimental design. hESC-derived TEC-like cells generated under shControl or shHOXA3 conditions were co-cultured with human umbilical cord blood-derived CD34^+^ HPCs for 7 days, followed by flow cytometric analysis. (**B**) Representative flow cytometry plots of CD45 and CD3 expression showing the CD45^+^CD3^+^ population in the indicated co-cultures. (**C**) Quantification of the percentages of CD45^+^CD3^+^ cells across three independent experiments. Data are presented as mean ± SEM. Statistical significance was determined using an unpaired, two-tailed Student’s *t*-test. **** *p* < 0.0001 versus shControl.

Having observed impaired acquisition of thymic epithelial-associated features following HOXA3 knockdown, we next examined whether this alteration affected the early T-lineage-supporting capacity of the derived TEC-like cells. Day 13 hESC-derived TEC-like cultures from shControl or shHOXA3 conditions were co-cultured with CD34^+^ hematopoietic progenitor cells (HPCs) isolated from human umbilical cord blood for one week, followed by flow cytometric analysis (Figure 3A). The CD45^+^CD3^+^ population was used as an early T-lineage-associated readout. A distinct CD45^+^CD3^+^ population was detected in co-cultures supported by shControl TEC-like cells (Figure 3B), whereas the proportion of CD45^+^CD3^+^ cells was markedly reduced in co-cultures containing shHOXA3 TEC-like cells (Figure 3B). Quantification across three biological replicates confirmed a significant decrease in the percentage of CD45^+^CD3^+^ cells in the shHOXA3 group compared with the shControl group (Figure 3C). These results are consistent with reduced early T-lineage-supporting capacity after HOXA3 knockdown.

### 3.4. HOXA3 Knockdown Induces Broad Transcriptomic Remodeling in hESC-Derived TEC-like Cells

**Figure 4:**
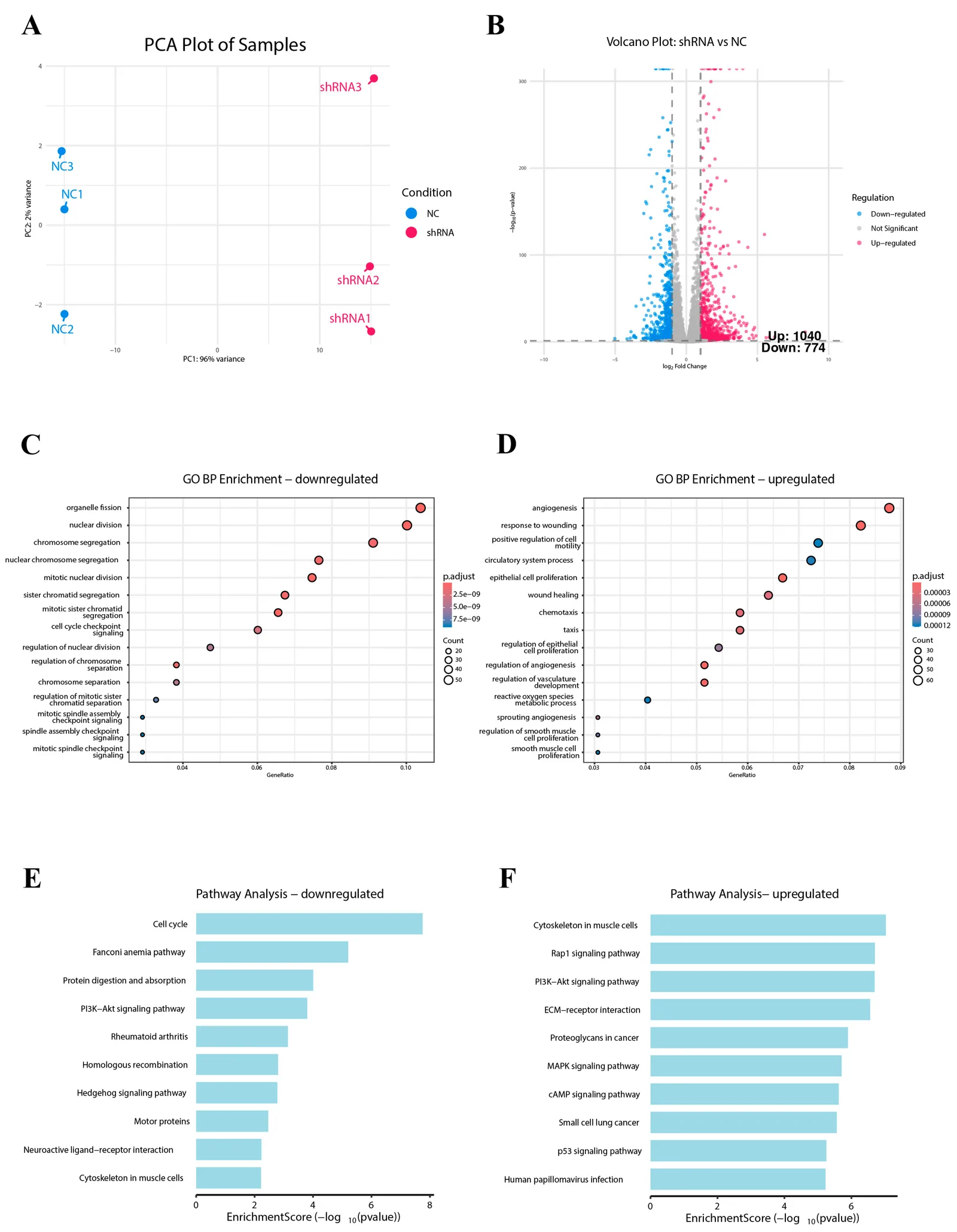
HOXA3 knockdown induces broad transcriptomic remodeling in hESC-derived TEC-like cells. (**A**) Principal component analysis (PCA) of bulk RNA-sequencing profiles from day 13 shControl and shHOXA3 TEC-like cultures, with three independent biological replicates per group. PC1 and PC2 accounted for 96% and 2% of the total variance, respectively. (**B**) Volcano plot showing differentially expressed genes (DEGs) in shHOXA3 TEC-like cells relative to shControl cells. Genes with an adjusted *p* value < 0.05 and |log_2_ fold change| ≥ 1 were considered significantly differentially expressed. Significantly upregulated genes (*n* = 1040) are shown in magenta, significantly downregulated genes (*n* = 774) in blue, and non-significant genes in gray. (**C**,**D**) Gene Ontology (GO) biological process enrichment analysis of genes significantly downregulated (**C**) or upregulated (**D**) following HOXA3 knockdown. The horizontal axis indicates the gene ratio, circle size represents the number of genes associated with each term, and color indicates the adjusted *p* value. (**E**,**F**) KEGG pathway enrichment analysis of genes significantly downregulated (**E**) or upregulated (**F**) following HOXA3 knockdown. Bar length represents the enrichment score for each pathway.

To define the transcriptional consequences of HOXA3 knockdown during thymic epithelial differentiation, we performed bulk RNA sequencing of day 13 shControl and shHOXA3 cultures, with three independent biological replicates per group. Principal component analysis (PCA) revealed clear segregation between the two groups, with the first principal component accounting for 96% of the total variance (Figure 4A). Given the large proportion of variance explained by PC1, this separation was interpreted as reflecting broad transcriptional differences between the two experimental conditions rather than changes in a single HOXA3-dependent pathway. Differential expression analysis identified a total of 1814 differentially expressed genes using an adjusted *p* value < 0.05 and |log_2_ fold change| > 1 as the significance thresholds. Of these, 1040 genes were upregulated and 774 were downregulated in shHOXA3 TEC-like cells relative to shControl cells (Figure 4B). Together, these results indicate broad transcriptomic differences associated with HOXA3 knockdown in developing TEC-like cells.

To characterize the biological programs associated with these transcriptional changes, Gene Ontology (GO) biological process enrichment analysis was performed separately for the upregulated and downregulated gene sets in shHOXA3 cells relative to shControl cells. Genes downregulated following HOXA3 knockdown were predominantly enriched in cell-cycle and chromosome-maintenance processes, including organelle fission, nuclear division, chromosome segregation, mitotic nuclear division, and sister chromatid segregation (Figure 4C). In contrast, upregulated genes were enriched in processes associated with responses to wounding, cell motility, chemotaxis, angiogenesis, and tissue remodeling (Figure 4D). KEGG pathway enrichment analysis further reinforced these distinct molecular signatures. Downregulated genes were prominently associated with the cell-cycle, Fanconi anemia, homologous recombination, and Hedgehog signaling pathways (Figure 4E). Upregulated genes were enriched in pathways including Rap1 signaling, ECM–receptor interaction, MAPK signaling, cAMP signaling, and p53 signaling (Figure 4F). These transcriptomic data revealed that HOXA3 knockdown induced extensive molecular alterations during the 3PPE-to-TEC transition, consistent with the impaired thymic epithelial differentiation and T-cell-supporting phenotype observed above.

Therefore, these findings demonstrate that HOXA3 knockdown broadly remodels the transcriptional state of developing TEC-like cells, characterized by the attenuation of cell-cycle and chromosome-maintenance programs and the induction of motility-, stress-response-, angiogenesis-, and tissue-remodeling-associated programs. We therefore examined the differentially expressed genes for candidate stromal mediators that could link HOXA3-regulated thymic epithelial programs to T-cell-supporting function.

### 3.5. HOXA3 Knockdown Reduces CXCL12 Expression in hESC-Derived TEC-like Cells

**Figure 5:**
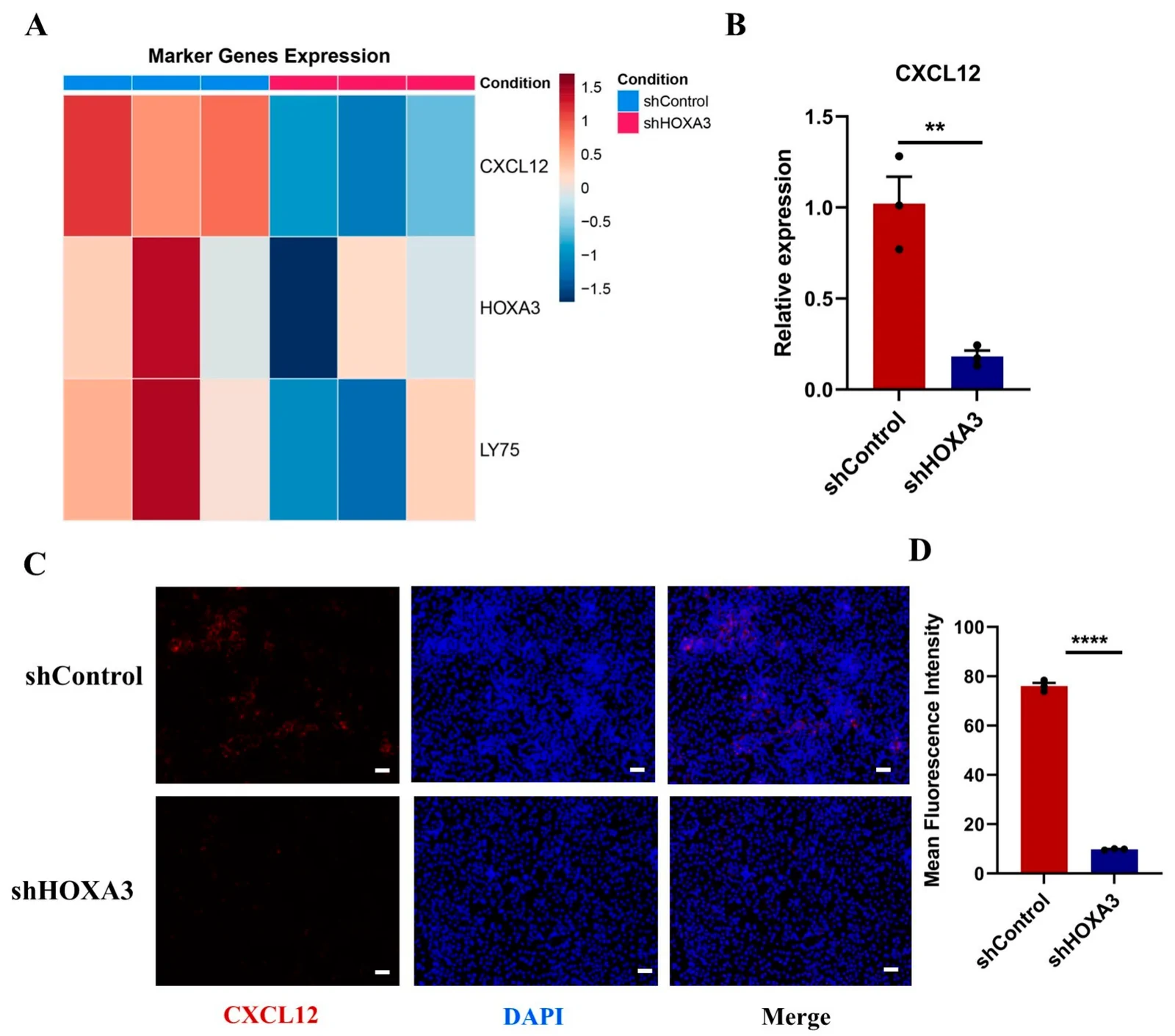
HOXA3 knockdown reduces CXCL12 expression in hESC-derived TEC-like cells. (**A**) Heatmap showing scaled expression levels of selected genes, including *CXCL12*, *HOXA3*, and *LY75*, in bulk RNA-sequencing data from shControl and shHOXA3 TEC-like cell cultures. Each group included three independent biological replicates. Red and blue indicate relatively higher and lower expression, respectively. (**B**) RT-qPCR validation of *CXCL12* transcript levels in shControl and shHOXA3 cultures. (**C**) Representative immunofluorescence images showing CXCL12 expression in shControl and shHOXA3 cultures. CXCL12 is shown in red, and nuclei were counterstained with DAPI in blue. Scale bars, 50 μm. (**D**) Quantification of CXCL12 mean fluorescence intensity in shControl and shHOXA3 cultures. Data in (**B**,**D**) are presented as mean ± SEM from three independent experiments. Statistical significance was determined using an unpaired, two-tailed Student’s *t*-test. ** *p* < 0.01 and **** *p* < 0.0001 versus shControl.

Among the differentially expressed genes identified following HOXA3 knockdown, CXCL12 was notably downregulated in shHOXA3 TEC-like cells (Figure 5A). Because CXCL12 is a thymic stromal chemokine with established roles in progenitor localization and intrathymic T-cell development [19,20], we selected CXCL12 for further validation as a candidate factor linking HOXA3-associated transcriptional changes to T-cell-supporting function. RT-qPCR confirmed a significant reduction in CXCL12 transcript levels in shHOXA3 cultures compared with shControl cultures (Figure 5B). Consistent with the transcriptional result, immunofluorescence staining showed markedly reduced CXCL12 protein expression following HOXA3 knockdown (Figure 5C). Quantification of mean fluorescence intensity across three independent experiments further confirmed the significant decrease in CXCL12 protein abundance in shHOXA3 cultures (Figure 5D). These findings identify CXCL12 as a HOXA3-responsive gene in hESC-derived TEC-like cells and suggest that its reduced expression may contribute to the impaired T-cell-supporting phenotype observed following HOXA3 knockdown. We therefore next examined whether HOXA3 occupies the CXCL12 regulatory region and whether CXCL12 supplementation could improve the T-cell-supporting capacity of HOXA3-knockdown TEC-like cells.

### 3.6. HOXA3 Occupies the CXCL12 Regulatory Region in hESC-Derived TEC-like Cells

**Figure 6:**
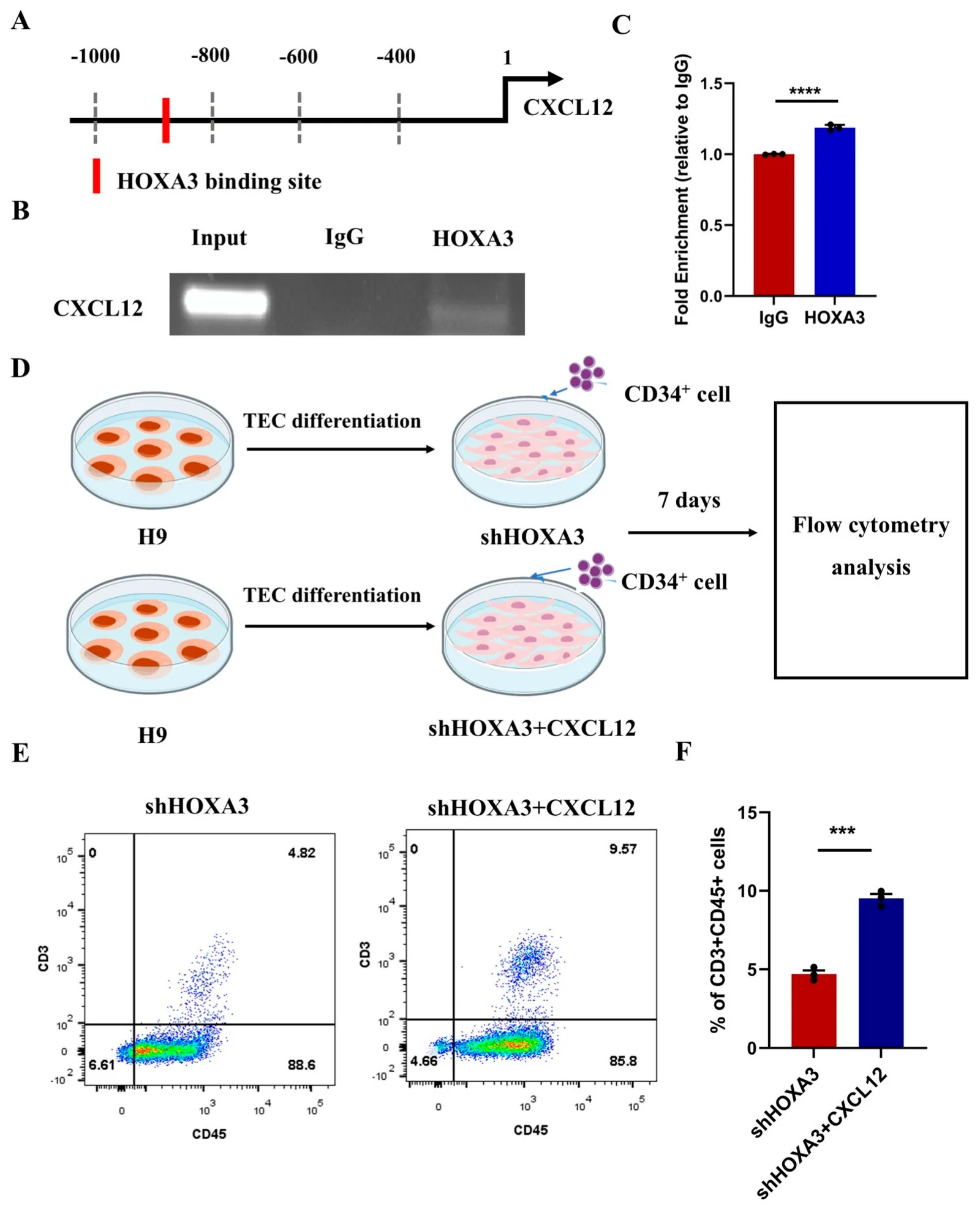
HOXA3 ChIP enriches a predicted CXCL12 regulatory region, and CXCL12 supplementation partially increases early T-lineage-associated output in HOXA3-knockdown co-cultures. (**A**) Schematic representation of the upstream regulatory region of the human *CXCL12* gene, showing a predicted HOXA3-binding site relative to the transcription start site (+1). (**B**) Representative ChIP-PCR analysis of the *CXCL12* regulatory region in hESC-derived TEC-like cells. Chromatin was immunoprecipitated using an anti-HOXA3 antibody or an isotype-matched control IgG, followed by PCR amplification of the indicated *CXCL12* regulatory fragment. Input chromatin is shown as a positive control. (**C**) Densitometric quantification of ChIP-PCR products from three independent ChIP experiments. Enrichment of the *CXCL12* regulatory fragment in the HOXA3 immunoprecipitate was expressed relative to the IgG control, which was set to 1.0. **** *p* < 0.0001 versus the IgG control. (**D**) Schematic illustration of the CXCL12 supplementation experiment. HOXA3-knockdown TEC-like cells were co-cultured with human umbilical cord blood-derived CD34^+^ HPCs for 7 days in the absence or presence of recombinant human CXCL12, followed by flow cytometric analysis. (**E**) Representative flow cytometry plots of CD45 and CD3 expression showing the CD45^+^CD3^+^ population in untreated shHOXA3 and CXCL12-supplemented shHOXA3 co-cultures. (**F**) Quantification of the percentages of CD45^+^CD3^+^ cells across three independent experiments. Data are presented as mean ± SEM. *** *p* < 0.001 versus untreated shHOXA3 co-cultures.

To examine whether HOXA3 is associated with a candidate regulatory region of CXCL12, we analyzed the upstream sequence of CXCL12 and identified a putative HOXA3-binding motif within a predicted regulatory region (Figure 6A). Chromatin immunoprecipitation (ChIP) was then performed in hESC-derived TEC-like cells using an anti-HOXA3 antibody or an isotype-matched control IgG as a negative control. PCR amplification of the immunoprecipitated chromatin revealed enrichment of the CXCL12 regulatory fragment in the HOXA3 immunoprecipitate compared with the IgG control (Figure 6B). Quantification of ChIP-PCR products across three biological replicates showed approximately 1.5-fold enrichment relative to the IgG control (Figure 6C). Together with the reduction in CXCL12 mRNA and protein after HOXA3 knockdown, these findings support HOXA3 association with the predicted regulatory region and are consistent with CXCL12 expression being responsive to the HOXA3-knockdown condition.

### 3.7. CXCL12 Supplementation Partially Increases CD45^+^CD3^+^ Early T-Lineage Output in HOXA3-Knockdown Co-Cultures

To test whether CXCL12 can contribute to the reduced early T-lineage-associated output observed in the HOXA3-knockdown condition, recombinant CXCL12 was added to co-cultures of shHOXA3 TEC-like cells and cord blood CD34^+^ hematopoietic progenitor cells, with untreated shHOXA3 co-cultures serving as controls (Figure 6D). After one week, flow cytometric analysis showed that CXCL12 supplementation increased the proportion of CD45^+^CD3^+^ cells compared with untreated shHOXA3 co-cultures (Figure 6E). Quantification across three biological replicates confirmed this increase in CD45^+^CD3^+^ cell generation following CXCL12 supplementation (Figure 6F). Thus, these results support a contributory role for CXCL12.

## 4. Discussion

In this study, we used an hESC-based differentiation system to examine HOXA3 knockdown during the transition from 3PPE toward a thymic epithelial-like lineage. HOXA3 knockdown reduced thymic epithelial-associated features and induced broad transcriptomic remodeling in developing TEC-like cells. In the co-culture assay, the HOXA3-knockdown condition was associated with a markedly lower proportion of CD45^+^CD3^+^ cells from human CD34^+^ hematopoietic progenitors. CXCL12 expression was reduced at both transcript and protein levels following HOXA3 knockdown. Conventional ChIP-PCR showed enrichment of a predicted CXCL12 regulatory region in HOXA3 im-munoprecipitates, and recombinant CXCL12 partially increased CD45^+^CD3^+^ output. Taken together, the data support involvement of HOXA3 in this hESC-derived thymic epithelial-like differentiation model and a functional contribution of CXCL12.

Our three-stage differentiation system recapitulates sequential developmental transitions from definitive endoderm to 3PPE and subsequently toward the thymic epithelial-like lineage [16], providing a stage-resolved context for examining HOXA3 function during human TEC-like cell differentiation. Genetic studies in mice have established an essential role for HOXA3 in pharyngeal apparatus and thymus development [15], while our previous work demonstrated that HOXA3 regulates the generation of hESC-derived 3PPE through EPHB2-mediated Wnt signaling [16]. The present study extends these findings by showing that HOXA3 knockdown initiated after 3PPE specification impairs subsequent thymic epithelial-like cell differentiation. Reduced expression of K8, AIRE, and CD205, together with decreased FOXN1 protein, indicates the disruption of a broader thymic epithelial-associated program. The reduction in AIRE is consistent with impaired acquisition of AIRE-associated medullary epithelial programs relevant to central tolerance [22], whereas decreased FOXN1 points to disruption of a core transcriptional network required for thymic epithelial progenitor differentiation [22,23]. Bulk RNA sequencing further revealed extensive transcriptional remodeling, characterized by suppression of cell-cycle and chromosome-maintenance programs and induction of wound-response, motility, chemotaxis, angiogenesis, and tissue-remodeling programs. These changes suggest that HOXA3 loss shifts the cellular state of developing TEC-like cells during the 3PPE-to-TEC-like cell transition and are consistent with the altered morphology and reduced cell density observed after HOXA3 knockdown. However, because cell proliferation and viability were not directly examined, these pathway-level associations should be interpreted cautiously. Although the reduction in FOXN1 places HOXA3 within a broader TEC-like cell developmental regulatory network, the present data do not establish a direct regulatory relationship between HOXA3 and FOXN1.

A major functional phenotype associated with HOXA3 knockdown was the lower proportion of CD45^+^CD3^+^ cells after one week of co-culture with cord blood CD34^+^ hematopoietic progenitors. This result provides an early T-lineage-associated readout but does not demonstrate complete thymopoiesis, positive or negative selection, or mature T-cell generation, because additional developmental markers such as CD5, CD7, CD1a, CD4, CD8, and TCR were not assessed. The assay was performed in medium supplemented with SCF, IL-7, FLT3L and DLL4; a CD34^+^-only/no-stroma control was not included. Therefore, the current experiment does not quantify the absolute stromal contribution to CD45^+^CD3^+^ generation. Moreover, viable stromal-cell number was not re-quantified or normalized at co-culture initiation; thus, the lower CD45^+^CD3^+^ output in the shHOXA3 condition could reflect a combination of altered cell number, altered cellular state, and qualitative changes in stromal support. Baseline CD3 staining of the starting CD34^+^ fraction was also not included in the current dataset, so a contribution from rare mature-cell contamination cannot be completely excluded. These limitations substantially narrow the interpretation to facilitate early T-lineage differentiation in vitro, rather than demonstrating complete thymopoiesis.

CXCL12 provides a mechanistic and functional link between HOXA3 activity in developing TEC-like cells and their T-cell-supporting capacity. CXCL12, also known as stromal cell-derived factor 1 (SDF-1), is an important stromal chemokine that signals through CXCR4 to regulate thymic progenitor localization and intrathymic T-cell development [19,20]. In our system, HOXA3 knockdown reduced CXCL12 expression at both the transcript and protein levels. Motif analysis identified a putative HOXA3-binding site within a predicted CXCL12 regulatory region, and chromatin immunoprecipitation demonstrated enrichment of this region in HOXA3 immunoprecipitates relative to the IgG control. These data support association of HOXA3 with the candidate region but do not establish direct transcriptional activation. Functionally, recombinant CXCL12 increased CD45^+^CD3^+^ output in HOXA3-knockdown co-cultures, but the rescue was partial and necessity was not tested. Therefore, the data support a contribution of CXCL12 without demonstrating sufficiency, necessity, or predominant mediation. Quantitative ChIP-qPCR with positive and negative genomic controls, promoter-reporter assays, binding-site mutagenesis, and loss-of-function testing of CXCL12/CXCR4 would be required to define the regulatory mechanism more rigorously.

In this study, several limitations should be considered when interpreting these findings. Firstly, the study used a single H9/WA09 hESC line and a single shRNA sequence targeting HOXA3; therefore, line-to-line generalizability and sequence-specific off-target effects cannot be fully assessed without an independent hESC line, an independent shRNA, shRNA-resistant HOXA3 rescue, or complementary genome-editing approaches. Secondly, shHOXA3 cultures showed reduced cell density and suppression of cell-cycle-associated programs, but proliferation, apoptosis/viability, and viable stromal-cell number were not directly quantified; the observed changes may therefore reflect both lineage-associated effects and broader changes in cell abundance or state. Thirdly, the one-week cytokine- and DLL4-supplemented co-culture assay used CD45^+^CD3^+^ cells as the principal readout, without additional T-lineage markers, baseline CD3 assessment of the input CD34^+^ population, or a CD34^+^-only/no-stroma control. Finally, the ChIP experiment used conventional gel-based ChIP-PCR without quantitative ChIP-qPCR or positive/negative genomic control regions. Thus, the current data support an association of HOXA3 with a predicted CXCL12 regulatory region and a partial functional contribution of CXCL12, but do not establish direct transcriptional activation or an exclusive HOXA3-CXCL12 mechanism. Despite these limitations, this study provides a tractable hESC-based platform for investigating human thymic epithelial development and identifies CXCL12 as a functionally relevant downstream factor.

## 5. Conclusions

Using a stage-resolved hESC differentiation model, we found that HOXA3 knock-down during the 3PPE-to-thymic epithelial-like transition was associated with reduced acquisition of thymic epithelial-associated features, lower FOXN1 protein, broad transcriptomic remodeling, and diminished CD45^+^CD3^+^ early T-lineage output in co-culture. HOXA3 ChIP enriched a predicted CXCL12 regulatory region, HOXA3 knockdown reduced CXCL12 expression, and recombinant CXCL12 partially increased the functional readout. These findings support involvement of HOXA3 in this hESC-derived thymic epithelial-like differentiation model and identify CXCL12 as one contributing factor to the associated early T-lineage phenotype. Together, this hESC-based system provides a tractable platform for further investigation of developmental transcriptional programs and stromal functions relevant to early human T-lineage differentiation.

## Data Availability

The data that support the findings of this study are available from the corresponding author upon the request. The RNA-seq datasets have been deposited in GEO with accession number GSE344232 (https://www.ncbi.nlm.nih.gov/gds/?term=GSE344232, accessed on 24 August 2026).

## Supplementary Materials

The following supporting information can be downloaded at: https://www.mdpi.com/article/10.3390/cells15191764/s1, Table S1: Primers used for qRT-PCR analysis; Table S2: Antibodies used in this study; Table S3: Primers used for ChIP-PCR in this study.

## References

[1] Lopes N., Sergé A., Ferrier P., Irla M. (2015). Thymic Crosstalk Coordinates Medulla Organization and T-Cell Tolerance Induction. Front. Immunol..

[2] Gras-Peña R., Danzl N.M., Khosravi-Maharlooei M., Campbell S.R., Ruiz A.E., Parks C.A., Savage W.M.S., Holzl M.A., Chatterjee D., Sykes M. (2022). Human stem cell-derived thymic epithelial cells enhance human T-cell development in a xenogeneic thymus. J. Allergy Clin. Immunol..

[3] Luo Y., Wang W., Yan J. (2026). Thymic APC Networks Orchestrate T-Cell Selection: Mechanisms and Therapeutic Opportunities in Immune Disorders. Immunology.

[4] Klein L., Kyewski B., Allen P.M., Hogquist K.A. (2014). Positive and negative selection of the T cell repertoire: What thymocytes see (and don’t see). Nat. Rev. Immunol..

[5] Santamaria J.C., Irla M. (2026). Age-related thymic involution: Mechanistic insights and rejuvenating approaches to restore immune function. Sci. Adv..

[6] Palmer D.B. (2013). The Effect of Age on Thymic Function. Front. Immunol..

[7] Bosticardo M., Notarangelo L.D. (2023). Human thymus in health and disease: Recent advances in diagnosis and biology. Semin. Immunol..

[8] Parent A.V., Russ H.A., Khan I.S., LaFlam T.N., Metzger T.C., Anderson M.S., Hebrok M. (2013). Generation of Functional Thymic Epithelium from Human Embryonic Stem Cells that Supports Host T Cell Development. Cell Stem Cell.

[9] Sun X., Xu J., Lu H., Liu W., Miao Z., Sui X., Liu H., Su L., Du W., He Q. (2013). Directed Differentiation of Human Embryonic Stem Cells into Thymic Epithelial Progenitor-like Cells Reconstitutes the Thymic Microenvironment In Vivo. Cell Stem Cell.

[10] Soh C.L., Giudice A., Jenny R.A., Elliott D.A., Hatzistavrou T., Micallef S.J., Kianizad K., Seach N., Zúñiga-Pflücker J.C., Chidgey A.P. (2014). FOXN1^GFP/w^ Reporter hESCs Enable Identification of Integrin-β4, HLA-DR, and EpCAM as Markers of Human PSC-Derived FOXN1^+^ Thymic Epithelial Progenitors. Stem Cell Rep..

[11] Gordon J., Manley N.R. (2011). Mechanisms of Thymus Organogenesis and Morphogenesis. Development.

[12] Provin N., Giraud M. (2022). Differentiation of Pluripotent Stem Cells into Thymic Epithelial Cells and Generation of Thymic Organoids: Applications for Therapeutic Strategies Against APECED. Front. Immunol..

[13] Manley N.R., Capecchi M.R. (1995). The Role of Hoxa-3 in Mouse Thymus and Thyroid Development. Development.

[14] Chojnowski J.L., Masuda K., Trau H.A., Thomas K., Capecchi M., Manley N.R. (2014). Multiple Roles for HOXA3 in Regulating Thymus and Parathyroid Differentiation and Morphogenesis in Mouse. Development.

[15] Su D.M., Ellis S., Napier A., Lee K., Manley N.R. (2001). Hoxa3 and Pax1 Regulate Epithelial Cell Death and Proliferation during Thymus and Parathyroid Organogenesis. Dev. Biol..

[16] Fu Y., Zhang X., Wu H., Zhang P., Liu S., Guo T., Shan H., Liang Y., Chen H., Xie J. (2024). HOXA3 Functions as the On-off Switch to Regulate the Development of hESC-Derived Third Pharyngeal Pouch Endoderm Through EPHB2-Mediated Wnt Pathway. Front. Immunol..

[17] Manley N.R., Condie B.G., Liston A. (2010). Transcriptional Regulation of Thymus Organogenesis and Thymic Epithelial Cell Differentiation. Progress in Molecular Biology and Translational Science.

[18] Anderson G., Jenkinson E.J. (2001). Lymphostromal Interactions in Thymic Development and Function. Nat. Rev. Immunol..

[19] Plotkin J., Prockop S.E., Lepique A., Petrie H.T. (2003). Critical Role for CXCR4 Signaling in Progenitor Localization and T Cell Differentiation in the Postnatal Thymus. J. Immunol..

[20] Ara T., Itoi M., Kawabata K., Egawa T., Tokoyoda K., Sugiyama T., Fujii N., Amagai T., Nagasawa T. (2003). A Role of CXC Chemokine Ligand 12/Stromal Cell-Derived Factor-1/Pre-B Cell Growth Stimulating Factor and Its Receptor CXCR4 in Fetal and Adult T Cell Development in Vivo. J. Immunol..

[21] Romano R., Palamaro L., Fusco A., Giardino G., Gallo V., Del Vecchio L., Pignata C. (2013). FOXN1: A Master Regulator Gene of Thymic Epithelial Development Program. Front. Immunol..

[22] Kyewski B., Klein L. (2006). A Central Role for Central Tolerance. Annu. Rev. Immunol..

[23] Blackburn C.C., Augustine C.L., Li R., Harvey R.P., Malin M.A., Boyd R.L., Miller J.F., Morahan G. (1996). The nu Gene Acts Cell-Autonomously and is Required for Differentiation of Thymic Epithelial Progenitors. Proc. Natl. Acad. Sci. USA.

