# Supplementary material for: HOXA3 Knockdown Impairs hESC-Derived Thymic Epithelial-like Cell Differentiation and CXCL12-Associated Early T-Lineage Support
Source: Cells. 2026 Sep 27;15(19):1764. doi: 10.3390/cells15191764 (PMC13664202; doi:10.3390/cells15191764)
Supplement: Supplementary file 1 [file cells-15-01764-s001.zip › cells-4554704-supplementary.pdf]

Supplementary Information

HOXA3 knockdown impairs hESC-derived thymic epithelial-like cell differentiation and CXCL12-associated early T-lineage support

Huanhuan Shan[#1,2], Yingjie Fu[#1,2], Xinyi Shi[1,2], Sen Chen[1,2], Yuyou Duan*[1,2,3,4]

Affiliations:

[1]Laboratory of Stem Cells and Translational Medicine, Center for Medical Research on Innovation and Translation, Institute of Clinical Medicine, the Second Affiliated Hospital, School of Medicine, South China University of Technology; Guangzhou, 510006, China.

[2]Laboratory of Stem Cells and Translational Medicine, Institute for Life Science, School of Medicine, South China University of Technology; Guangzhou, 510006, China.

[3]National Engineering Research Center for Tissue Restoration and Reconstruction, South China University of Technology; Guangzhou, 510006, China.

[4]The Innovation Centre of Ministry of Education for Development and Diseases, the Second Affiliated Hospital, School of Medicine, South China University of Technology; Guangzhou 510006, China.

#: These authors contributed equally to this work.

* **Correspondence to** Yuyou Duan, Ph.D. (yuyouduan@scut.edu.cn)

Supplementary Table S1. Primers used for qRT-PCR analysis

Gene	Forward primer (5′→3′)	Reverse primer (5′→3′)
OCT4	AGGCAACCTGGAGAATTTGTTC	CACACTCGGACCACATCCTTC
SOX17	GGCGCAGCAGAATCCAGA	CCACGACTTGCCCAGCAT
CXCR4	AACCAGCGGTTACCATGGAG	CACGGAAACAGGGTTCCTTCA
PAX9	GGCGTGTGCGACAAGTAC	GGGCCAAGTTGCCGATCTT
SIX1	CTGCCGTCGTTTGGCTTTAC	GCTCTCGTTCTTGTGCAGGT
EPCAM	AATCGTCAATGCCAGTGTACTT	TCTCATCGCAGTCAGGATCATAA
KRT5	ATTCTGCTTCAAATCAGCCTTCA	AACTTTGGGTTCTCGTGTCAGC
KRT8	TCCTCAGGCAGCTATATGAAGAG	GGTTGGCAATATCCTCGTACTGT
FOXN1	GAGAGTGGTGCTGGGATGTT	ATGGGTTTTGGGAAGAGAGG
HOXA3	ATGCAAAAAGCGACCTACTACG	TGCAAAAAGCGACCTACTACG
AIRE	GCGGGAGAGGAGGTAAGAG	AGGACCCACACACAGTAGGG
CD205	GCAAGCGATACCTGTCACTCCT	ATAGGCAGTCCAGCGCAAACCA
CXCL12	ATTCTCAACACTCCAAACTGTGC	ACTTTAGCTTCGGGTCAATGC
GAPDH	GAAGATGGTGATGGGATTTC	GAAGGTGAAGGTCGGAGTC

Supplementary Table S2. Antibodies used in this study

Antigen	Source	Cat#
Keratin8	CST	4546s
AIRE	abcam	ab65040
FOXN1	SantaCruz	sc-271256
HOXA3	SantaCruz	sc-374237
HOXA3	SantaCruz	sc-374237X
CXCL12	R&D Systems	MAB350
GAPDH	Affinity Biosciences	AF7021
APC/Cy7 anti-human CD45	Biolegend	304014
Brilliant Violet 510™ anti-human CD3	Biolegend	317332
Anti-mouse IgG Fab2 Alexa Fluor (R) 488	CST	4408S
Anti-rabbit IgG Fab2 Alexa Fluor (R) 488	CST	4412S
Anti-mouse IgG Fab2 Alexa Fluor (R) 647	CST	4410S
HRP-labeled Goat Anti-Rabbit IgG(H+L)	Beyotime	A0208
HRP-labeled Goat Anti-Mouse IgG(H+L)	Beyotime	A0216

Supplementary Table S3. Primers used for ChIP-PCR in this study.

Target gene	Forward primer (5'→3')	Reverse primer (5'→3')	Amplicon (bp)	Position relative to TSS
CXCL12	ATCTCCCGTC CCACTCC	AGCGAATGTAG CCTTTGTACTT	84bp	-862 to-778
